# Dynamic molecular choreography induced by acute heat exposure in human males: a longitudinal multi-omics profiling study

**DOI:** 10.3389/fpubh.2024.1384544

**Published:** 2024-05-15

**Authors:** Jirui Wen, Juan Cheng, Ling Wang, Can Li, Yuhao Zou, Jiang Wu, Jifeng Liu

**Affiliations:** ^1^Department of Otolaryngology-Head and Neck Surgery, Deep Underground Space Medical Center, West China Hospital, Sichuan University, Guoxuexiang, Chengdu, China; ^2^Jinping Deep Underground Frontier Science and Dark Matter Key Laboratory of Sichuan Province, Liangshan, China; ^3^State Key Laboratory of Intelligent Construction and Healthy Operation and Maintenance of Deep Underground Engineering, Sichuan University, Chengdu, China; ^4^Med-X Center for Manufacturing, Sichuan University, Chengdu, China

**Keywords:** acute heat exposure, proteomics, metabolomics, molecular choreography, health

## Abstract

**Introduction:**

Extreme heat events caused by occupational exposure and heat waves are becoming more common. However, the molecular changes underlying the response to heat exposure in humans remain to be elucidated.

**Methods:**

This study used longitudinal multi-omics profiling to assess the impact of acute heat exposure (50°C for 30 min) in 24 subjects from a mine rescue team. Intravenous blood samples were collected before acute heat exposure (baseline) and at 5 min, 30 min, 1 h, and 24 h after acute heat exposure (recovery). In-depth multi-omics profiling was performed on each sample, including plasma proteomics (untargeted) and metabolomics (untargeted).

**Results:**

After data curation and annotation, the final dataset contained 2,473 analytes, including 478 proteins and 1995 metabolites. Time-series analysis unveiled an orchestrated molecular choreography of changes involving the immune response, coagulation, acid–base balance, oxidative stress, cytoskeleton, and energy metabolism. Further analysis through protein–protein interactions and network analysis revealed potential regulators of acute heat exposure. Moreover, novel blood-based analytes that predicted change in cardiopulmonary function after acute heat exposure were identified.

**Conclusion:**

This study provided a comprehensive investigation of the dynamic molecular changes that underlie the complex physiological processes that occur in human males who undergo heat exposure. Our findings will help health impact assessment of extreme high temperature and inspire future mechanistic and clinical studies.

## 1 Introduction

Prolonged exposure to extreme high temperatures in various industries, including steelmaking, tile production, mining, and rescue operations, can result in harmful health impact (1). Heat exposure can lead to heat-related illnesses encompassing a spectrum of syndromes like heat edema, muscle cramps, heat exhaustion and heatstroke (2, 3). Notably, numerous studies have highlighted moisture and heat as the most perceived adverse factors in deep underground work environments (4–6). For instance, Hunt et al. found that 79% of underground mine workers across three mine sites in northern Australia experienced symptoms of heat illness (7). Additionally, an analysis of hazards faced by mine rescuers revealed that the challenging mine environment led to body overheating and heatstroke, which accounted for 26% of total fatalities (8). Furthermore, several studies have demonstrated that the extreme heat environment places significant physiological and thermoregulatory stresses on firefighters, leading to stress, overexertion, and even death (9–11). In addition to occupational exposure, the general population is also vulnerable to extreme high temperature exposure due to the effects of climate change, including global warming (12). In 2019, heat waves in China resulted in approximately 26,800 deaths (13). Consequently, these studies have sparked significant concerns regarding the potential health impacts of extreme high temperatures on populations that are regularly exposed to heat.

The previous studies primarily concentrated on detailing the phenotypic and physiological reactions linked to heat exposure. Within the deep mine, miners sustained a core body temperature of 38°C throughout most of the day, leading to a notable rise in heart rate and severe dehydration (14, 15). Similarly, firefighters working in hot environments experienced elevated core body temperature, skin temperature, fluid intake, and heart rate (16–18). Another study showed that prolonged heat overload in firefighters working in hot environments increased arterial stiffness and vasodilation (19). These findings highlight the importance of assessing tissue and organ damage and anticipating potential long-term complications after heat exposure. It is crucial to note that delayed monitoring of core temperature or other physiological symptoms may lead to unexpected instances of severe heat-related conditions, such as heatstroke.

Molecular responses induced by extreme high temperatures can provide valuable indications of the physiological and metabolic changes that occur under such conditions. By monitoring these molecular responses, we can gain a better understanding of the body’s adaptive capacity to extreme high temperature and assess the potential degree of damage before clear clinical signs of thermal injury emerge. For example, analysis of blood mononuclear gene expression patterns in individuals exposed to extreme heat in a sauna (temperature of 75.7 ± 0.86°C) showed rapid changes in gene expression without a significant increase in core temperature. The reprogrammed transcriptome was mainly inhibitory, as genes involved in protein synthesis, mitochondrial bioenergetics, and immune function showing reduced expression (20). In another study by Bouchama et al. (21) the whole genome transcriptome in peripheral blood mononuclear cells of an adult cohort with heatstroke was examined. It was found that in heatstroke, the heat shock response was robust but failed to restore homeostasis due to proteotoxicity and a reduction in energy production (21). A meta-analysis of public gene expression database from human and mouse samples identified previously overlooked genes responding to heat, such as ABHD3, ZFAND2A, and USPL1 (22). However, knowledge of the response to heat exposure is limited to a small range of molecules and biological processes. This highlights the critical need of a systematic exploration of molecular dynamics in individuals exposed to extreme high temperature environments. In this study, we hypothesize that after heat exposure, the molecules undergo dynamic changes with distinct trends. While some molecules show reversible alterations, others do not. These diverse trends partly reflect the body’s adaptive capacity and the extent of damage sustained.

The objective of this study was to perform longitudinal multi-omics profiling of blood components from 24 mine rescuers, before and after a 30 min stay in a simulated extreme high temperature environment (50°C). The goal was to understand the short-term tolerance of humans to extreme high temperature and characterize the detailed series of events that occur in response to acute heat exposure. Our findings revealed an orchestrated molecular choreography of changes involving numerous molecules and pathways, and demonstrate the potential of multi-omics analytes application in monitoring recovery and predict complications following heat exposure. This study provides a comprehensive overview of the physiological processes that occur in humans who undergo extreme high temperature exposure and will inspire novel prevention strategies to reduce organ damage and long-term sequelae.

## 2 Methods

### 2.1 Study subjects

In May 2022, 24 participants were enlisted from the National Mine Emergency Rescue Furong Team (China) for this study. The study protocol received approval from the Ethics Review Committee (IRB number, 1351) of West China Hospital of Sichuan University. Prior to engaging in any study procedures, all participants provided written informed consent. A health questionnaire was used to screen for contraindications and/or comorbidities that might have prevented their exposure to extreme high temperatures. None of the participants had a history of underlying cardiovascular disease, cancer, or other chronic illnesses, and none had undergone heat or other stress training within 3 months prior to the study commencement.

### 2.2 Study design

In a systematic review assessing the impact of occupational heat strain, the studies included in the analysis reported a wide range of wet-bulb globe temperature (WBGT, 19.3 to 52.0°C) and air temperature (21.2–150.0°C, this extreme value was observed in a steel plant worksite) (23). Additionally, the rising global temperatures are pushing the human to endure extreme heat, with temperatures even reaching up to 54°C. As a balance between the intention of extreme high temperature exposure investigation and security considerations, we specifically chose 50°C as the environmental exposure temperature. Regarding the exposure time, a duration of 30 min was used based on the previous study that employed passive whole-body heat exposure (24–26). The research comprised three phases: baseline, exposure, and recovery. The baseline phase spanned from 09:00 am on the day prior to exposure until the day of exposure. The exposure phase occurred between 09:00 and 09:30 am. The recovery phase extended from 09:30 am until 09:30 am the following morning.

#### 2.2.1 Baseline period

Subjects’ physical condition was assessed on the day preceding exposure. Oral temperature was measured using a liquid crystal thermometer (Watermark^TM^). Grip strength of both hands was assessed using a grip dynamometer (EH 101, CAMRY). Objective fatigue was measured using performance tasks (BD-V-302A, BD-V-509A, Beda Qingniao). Pulmonary function was measured using spirometry. Heart function was examined with Doppler echocardiography. Urine specific gravity and pH were evaluated with urinalysis. Blood samples were collected at 07:30 am on the day of exposure.

#### 2.2.2 Exposure period

At 09:00 am on the day of exposure, participants were subjected to a high-temperature environment of 50°C and 37–40% relative humidity for 30 min. This was achieved using a mine roadway simulating test device designed to replicate fire conditions and aid in the training of the National Mine Emergency Rescue Team. Industrial heaters (JH-H150F, Cameron, China) were positioned at both ends of the roadway to generate the heat. Throughout the acute heat exposure, changes in heart rate were monitored using an electrocardiogram patch.

#### 2.2.3 Recovery period

Immediately after the heat exposure, oral temperature, grip strength, objective fatigue, subjective fatigue score, lung function, color Doppler echocardiography, urine samples were detected.

### 2.3 Objective fatigue

To measure the effects of heat exposure on fatigue, subjects were asked to perform two tasks. First, subjects participated in a bimanual interaction technique, where they controlled a cursor on a computer screen with a joystick using both hands, and the number of cursor-trajectory errors were recorded. Next, subjects participated in a visual reaction time test where they had to press color buttons as soon as corresponding-colored icons appeared on a screen using both hands. Reaction times were recorded.

### 2.4 Cardiopulmonary function tests

To assess the pulmonary function of subjects, we conducted a specific assay which measured Forced Expiratory Volume in One Second (FEV 1), Forced Vital Capacity (FVC), Vital Capacity (VC), Maximum Voluntary Ventilation (MVV), Maximum Mid-Respiratory Flow (MMF), and Maximum Expiratory Flow at 50% of Vital Capacity (V50). To assess the cardiac function of subjects, Echocardiograms were performed by experienced cardiologists using GE Vivid E9 and E9-S5 probes (1.6–3.2 MHz). According to international consensus guidelines, post-exposure images were obtained immediately after heat exposure, and imaging was satisfactory for all subjects. Echocardiography was analyzed according to the American Society of Echocardiography (ASE) guidelines (2015). Left ventricular function was quantified with the Simpson method using measurements of left ventricular ejection fraction (LVEF), stroke output (SV) and fractional shortening (FS). For statistical analysis of these parameters, Wilcoxon Signed Rank Test was performed as these quantitative data did not adhere to a normal distribution.

### 2.5 Blood collection and sample preparation

Following a 12-h overnight fast, blood samples were obtained from a vein in the upper forearm at baseline, 5 min, 30 min, and 1 h post-heat exposure, as well as the morning after heat exposure. The blood samples were collected in purple top vacutainers (BD), chilled on ice, and promptly processed. Subsequently, the blood samples were centrifuged at 3000 rpm for 10 min at 4°C. The top layer of EDTA plasma was removed, divided into 8 equal portions, and immediately frozen at −80°C. All blood samples were subjected to multi-level molecular profiling. An untargeted proteomics (proteins) and metabolomics (metabolites) approach was used to analyze the EDTA plasma samples.

### 2.6 Untargeted proteomics and data processing

Untargeted proteomics used Data independent acquisition (DIA). After the protein extraction from the plasma samples, protein concentration was determined using a BCA protein assay kit. Subsequently, 200 micrograms of each sample was subjected to 120 μL reducing buffer (10 mM DTT, 8 M Urea, 100 mM TEAB, pH 8.0). The solution was incubated at 60°C for 1 h, and IAA was added to the solution for 40 min at room temperature. Then the solutions were centrifuged on the filters at 12,000 rpm for 20 min at 4°C, followed by extensive washing. The purified samples were then subjected to overnight trypsin digestion at 37°C, resulting in the collection of peptides as filtrate. To facilitate further analysis, the peptide mixture was labeled using the TMT Tag Labeling kit. Then, all analyses were performed by a Q-Exactive HF mass spectrometer (Thermo, United States) equipped with a Nanospray Flex source (Thermo, United States). For the subsequent database search, Proteome Discoverer (v.2.4) was utilized to thoroughly search all raw data against the sample protein database. The database search was performed with Trypsin digestion specificity, considering alkylation on cysteine as fixed modifications. To ensure reliable results, a global false discovery rate (FDR) of 0.01 was set, and protein groups were considered for quantification only if they contained at least 2 peptides.

### 2.7 Untargeted metabolomics from plasma by liquid chromatography (LC)-MS

Plasma samples were prepared and analyzed in a random sequence. Progenesis QI software (v2.3, Non-linear Dynamics) was used to independently analyze data from each mode. Main parameters of 5 ppm precursor tolerance, 10 ppm product tolerance, and 5% product ion threshold were applied for qualitative analysis. Compound identification was conducted using databases such as The Human Metabolome Database (HMDB), Lipidmaps (V2.3), Metlin, EMDB, and PMDB, as well as self-built databases. A data matrix was created from positive and negative ion data and imported into R for Principle Component Analysis (PCA) to observe overall sample distribution and analysis stability. Orthogonal Partial Least-Squares-Discriminant Analysis (OPLS-DA) and Partial Least-Squares-Discriminant Analysis (PLS-DA) were used to distinguish metabolites that differed between groups, with 7-fold cross-validation and 200 Response Permutation Testing (RPT) used to prevent overfitting. Variable Importance of Projection (VIP) values from the OPLS-DA model ranked the contribution of each variable to group discrimination, and a two-tailed Student’s *T*-test verified significant differences in metabolites between groups with VIP values greater than 1.0 and *p*-values less than 0.05.

### 2.8 Quantification and statistical analysis

#### 2.8.1 Differential expression analysis

Differential expression analysis was performed using the DESeq2 package, with a *q-*value <0.05 and a fold change >2 or fold change <0.5 set as the threshold for significant differential expression analytes. R (v 3.2.0) was employed to conduct hierarchical cluster analysis of the differential expression analytes, illustrating the expression pattern of analytes across different groups and samples.

#### 2.8.2 Pathway enrichment analysis

To identify enriched pathways, Ingenuity pathway analysis (IPA, QIAGEN) was used to assess differentially expressed plasma analytes. The significance of each pathway was determined using hypergeometric probability (one-sided), while Fisher exact test was used to determine enrichment for proteins and metabolites. The *p-*values were corrected for multiple comparisons using the Benjamini-Hochberg method, with FDRs of <0.05 considered significant for proteins or metabolites, respectively. Fold change was estimated for significant molecules using the median of fold change relative to baseline, median of beta coefficients, and median of max (if up) or min (if down) fold change relative to baseline.

#### 2.8.3 Pathway dynamic analysis

Differential expressed analytes (FDR <0.05) were identified, and STRING analysis was used to explore the protein–protein interaction network before and after acute heat exposure. In PPI, proteins are represented as nodes, and edges between nodes represent interactions.

#### 2.8.4 WGCNA analysis

WGCNA was applied to analyze the co-expression modules and key analytes related to hub molecules. An adjacency matrix was transformed into a topological overlap matrix (TOM). Modules were identified with hierarchical clustering (minModuleSize =30). Module eigengenes were calculated. Module eigengenes (ME) and module memberships (MM) were used to determine key modules associated with hub molecules. The ME is defined as the first principal component of a given module and provides a representative expression profile. MM measured the relationship of a gene with the module eigengenes and reflects eigengene connectivity. The functions of metabolites in the key modules were investigated via Kyoto Encyclopedia of Genes and Genomes (KEGG) pathway enrichment analysis.

#### 2.8.5 Fuzzy *c*-mean clustering

The data underwent log2-transformation and *Z*-score scaling before conducting fuzzy *c*-mean clustering with the ‘Mfuzz’ R package (v2.20.0). The ‘elbow’ method was utilized to determine the minimum centroid distance or a range of cluster numbers and to select the optimal number. Additionally, *t*-distributed stochastic neighbor embedding (tSNE) scatterplots were generated using the ‘Rtsne’ R package, with the parameters set as perplexity = 5 and theta = 0.05.

### 2.9 Enzyme-linked immunosorbent assay

Plasma samples collected at baseline and 5 min, 30 min, 1 h, and 24 h after heat exposure were immediately centrifuged at 3,000 g for 10 min. To validate the key regulators identified by multi-omics analysis, serum levels of VWF, PF4, THBS1, HSP90AB1, and MPO were measured using enzyme-linked immunosorbent assay (ELISA) kits (Mlbio, Shanghai, China), following the manufacturer’s instructions. One-Way Repeated Measures ANOVA was employed to investigate the variability of parameters across different time points.

## 3 Results

### 3.1 Cohort characteristics and research design

**Figure 1:**
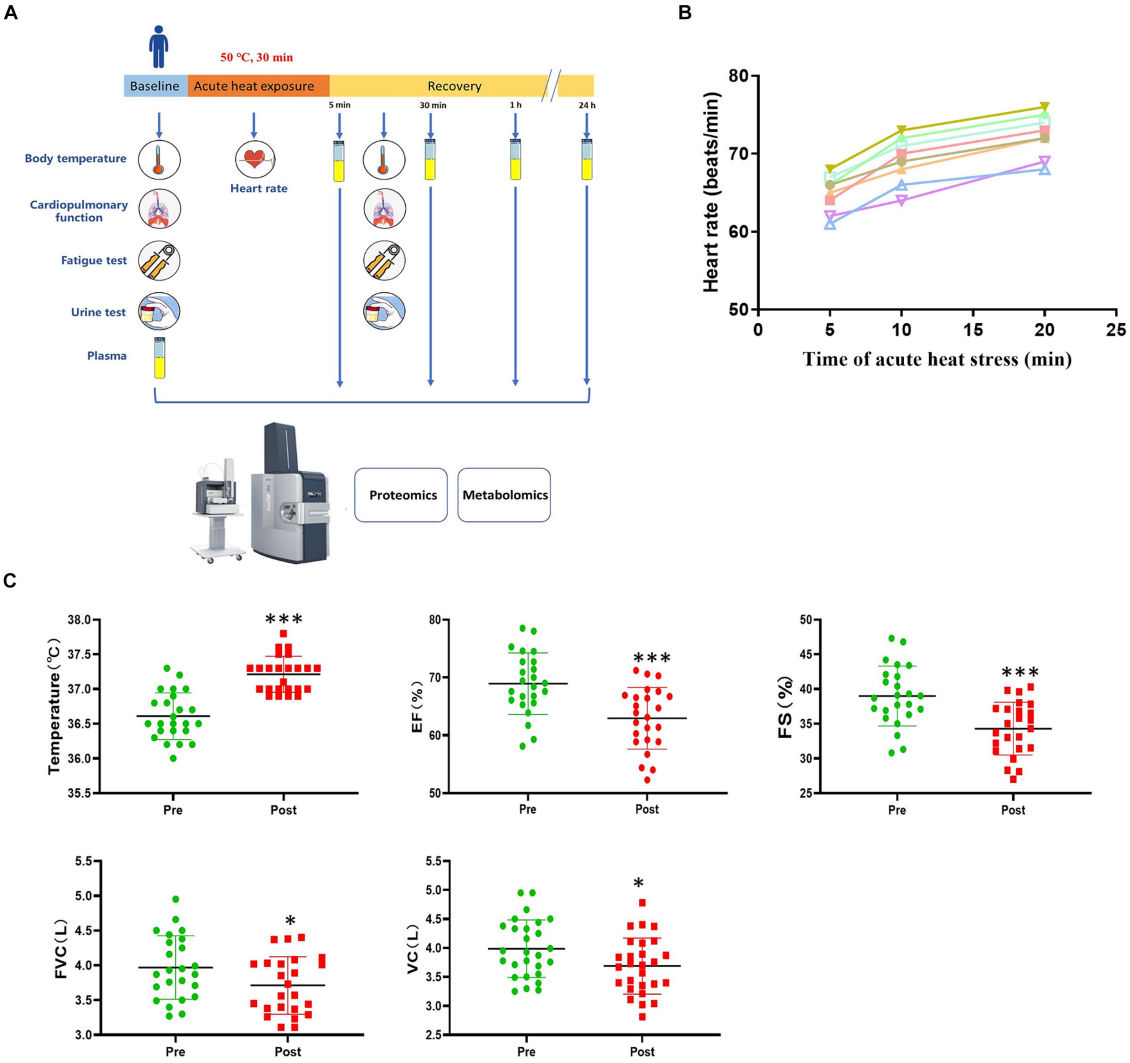
Study design and physiological response to acute heat exposure. **(A)** Overview of the study design. Subjects were exposed to an extreme high temperature environment of 50°C and 37–40% relative humidity for 30 min. Subjects underwent a physical examination before and after acute heat exposure. Intravenous blood samples were collected before acute heat exposure (baseline) and at 5 min, 30 min, 1 h, and 24 h after acute heat exposure (recovery) for the multi-omics analysis. **(B)** Heart rate changes were detected during 30 min high temperature environment simulation training. **(C)** Cardiopulmonary function changes in response to acute heat exposure. Core Temperature, the forced vital capacity (FVC), vital capacity (VC), left ventricular ejection fraction (EF), and fractional shortening (FS) were detected at pre- and post- acute heat exposure. * represents *p* < 0.05 and *** represents *p* < 0.001.

This study included 24 subjects. All were male, with a mean age of 32.9 years, mean height of 170.9 cm, and mean weight of 67.7 kg (Supplementary Table S1). The schematic diagram of experimental process is shown in Figure 1A. In-depth multi-omics profiling was performed, including plasma proteomics (untargeted) and metabolomics (untargeted). After data curation and annotation, the final dataset contained 2,473 analytes, including 478 proteins and 1995 metabolites.

During acute heat exposure, there was an observed increase in heart rate (Figure 1B). Immediately after acute heat exposure, oral temperature rose to 37.2°C compared to 36.6°C at baseline. Cardiopulmonary function parameters with significant changes were also shown. The forced vital capacity (FVC), vital capacity (VC), left ventricular ejection fraction (EF), and fractional shortening (FS) decreased after acute heat exposure (Figure 1C and Supplementary Table S2). For the urinalysis, the urine specific gravity of the participants had no change after acute heat exposure, while the urine PH value decreased (Supplementary Figure S1). As Supplementary Figure S2 shown, the fatigue increased after acute heat exposure.

### 3.2 Proteins and metabolites expression signature

**Figure 2:**
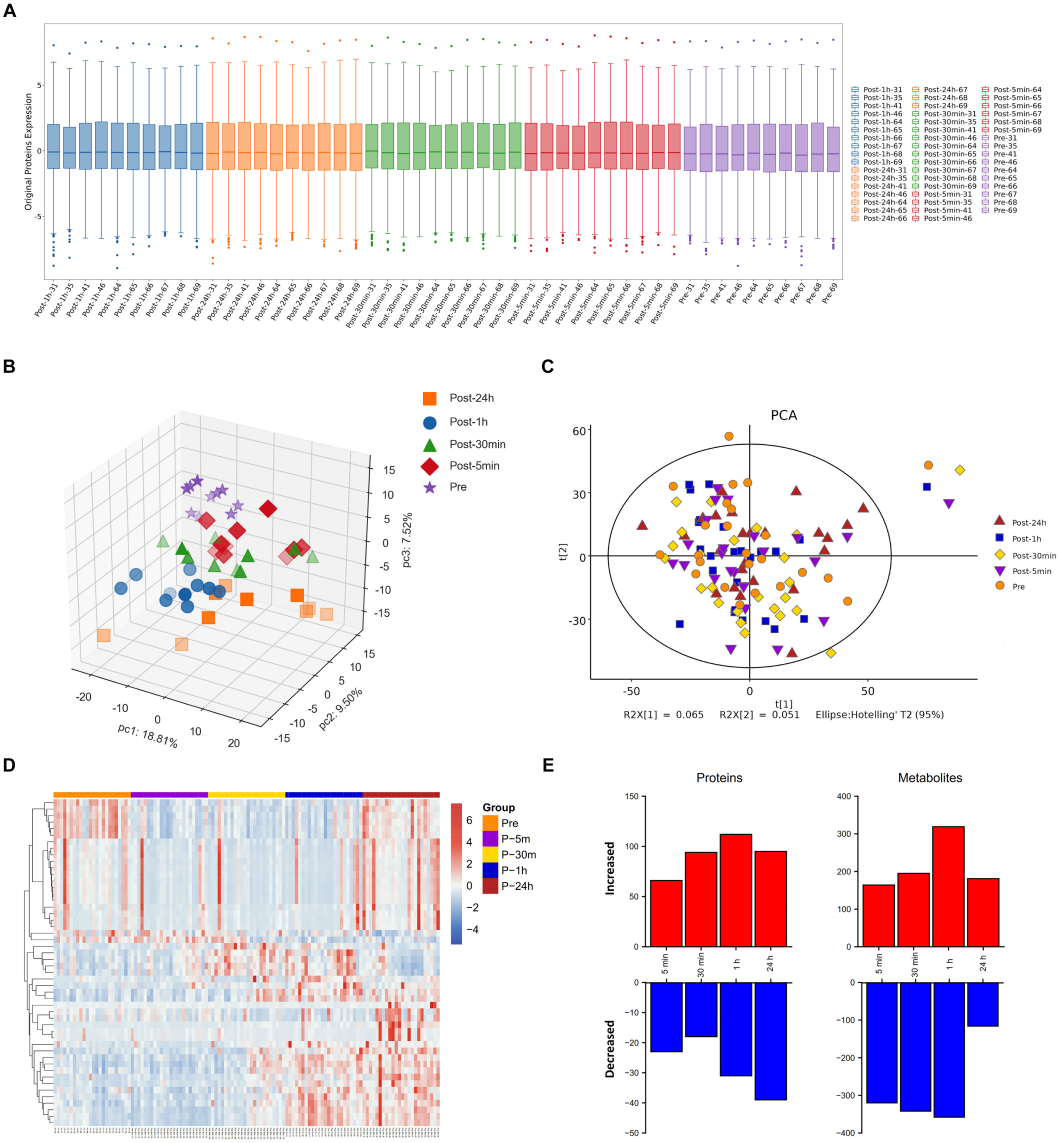
Molecular response to acute heat exposure. **(A)** Visualization display of credible protein expression distribution by boxplot. **(B,C)** Principal components analysis using proteomics **(B)** and metabolomics **(C)** data. **(D)** The heatmap showed the expression of differentially expressed metabolites. **(E)** Multi-omics analysis to explore changes in response to acute heat exposure across molecular layers at all the time points.

The boxplot reflected the expression patterns of proteins in each sample, indicating normal proteins expression patterns (Figure 2A). The omics datasets were assessed with principal component analysis, which suggested limited batch effects (Figures 2B,C). Additionally, the heatmap highlighted the expression of differentially expressed metabolites (Figure 2D). Exposure to an extreme high temperature environment of 50°C induced extensive changes in 2473 analytes spanning multiple omics layers, indicating system-wide changes (Figure 2E). Levels of circulating plasma proteins and metabolites were altered compared to baseline across all time points, and a large proportion of plasma proteins and metabolites remained significantly different from baseline after 24 h of recovery.

### 3.3 Functional analysis of differentially expressed proteins and metabolites at each time point

**Figure 3:**
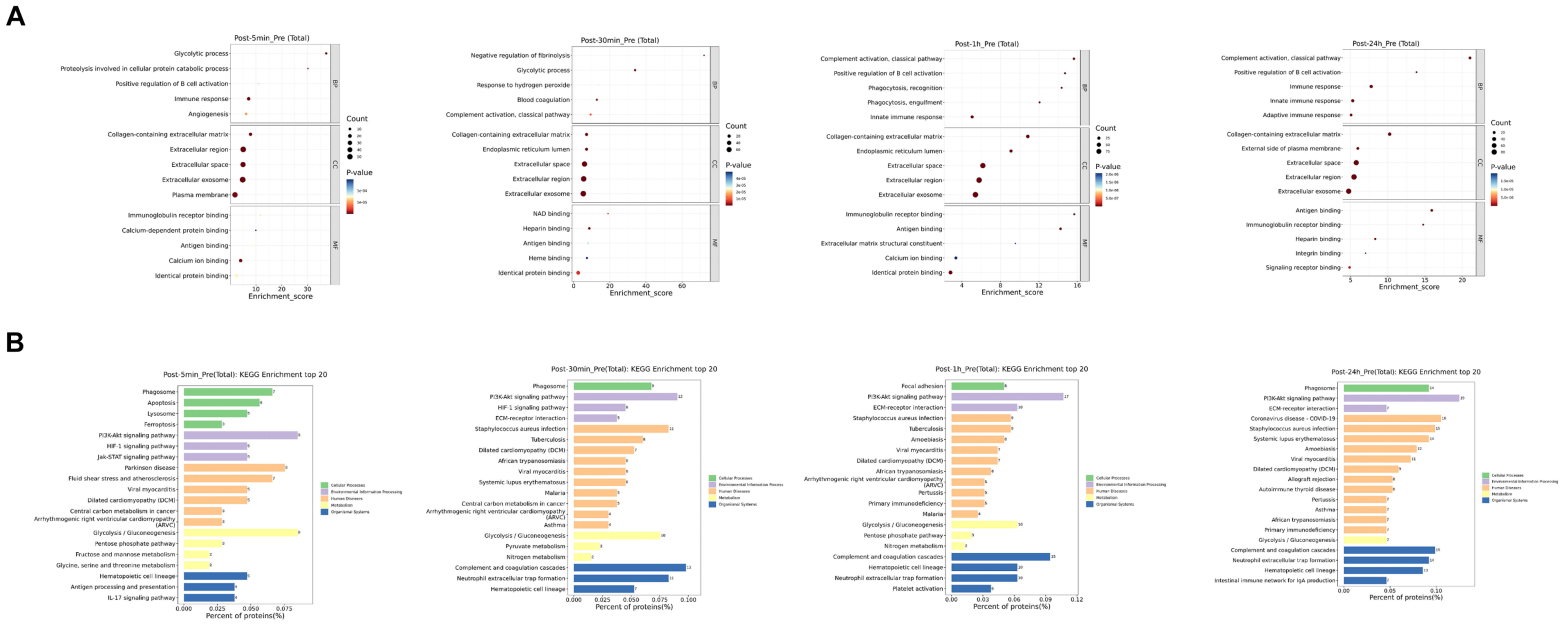
The enrichment analysis of protein changes in response to acute heat exposure. **(A)** Go enrichment analysis. The dot color represented pathway significance. The dot size represented number of proteins enriched in pathway. **(B)** KEGG enrichment analysis. Length represented the percent of proteins enriched in pathway.

**Figure 4:**
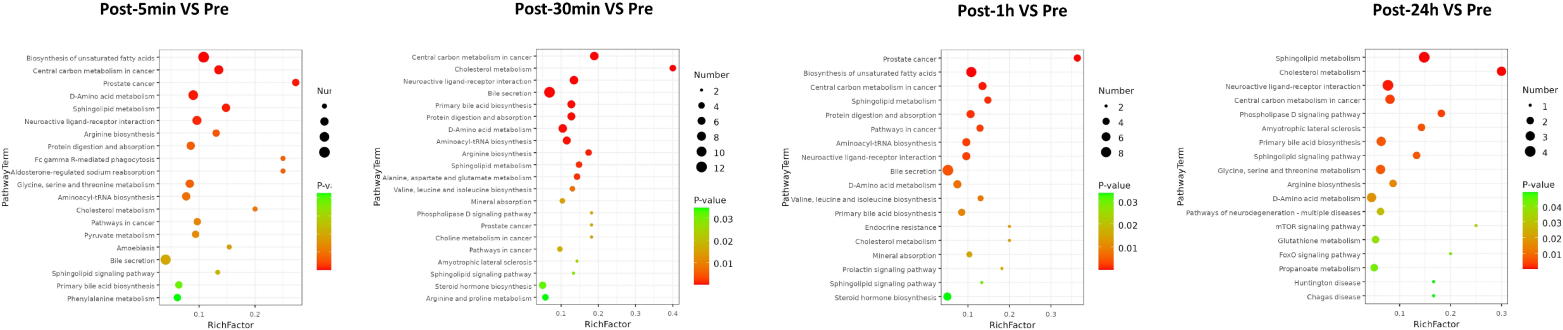
The enrichment analysis of metabolism changes in response to acute heat exposure. The dot color represented pathway significance in KEGG enrichment analysis. The dot size represented number of metabolites enriched in pathway.

As depicted in Figure 3, the biological processes that were altered after heat exposure primarily encompassed immune response, complement activation, extracellular region, ECM-receptor interaction, and glycolysis. Similarly, as illustrated in Figure 4, the metabolic pathways that were affected by heat exposure predominantly involved biosynthesis of unsaturated fatty acids, protein digestion and absorption, amino acid metabolism, cholesterol metabolism, and sphingolipid metabolism. These findings indicated substantial molecular reactions following acute heat exposure, emphasizing the need for further investigation into the temporal dynamics of these molecular responses.

### 3.4 Time series system-wide proteomics data

**Figure 5:**
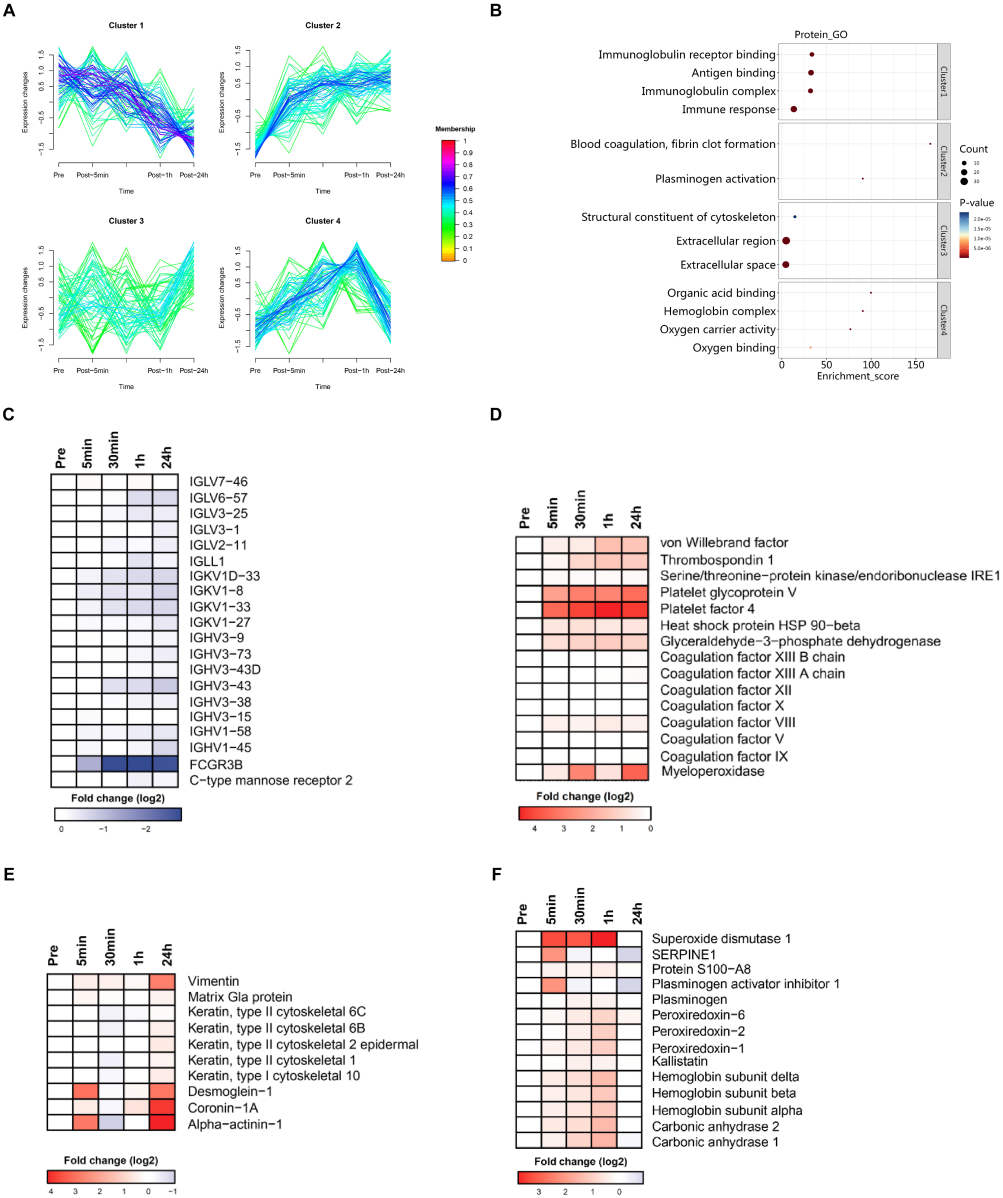
Time series system-wide proteomics data. **(A)** Clustering of longitudinal trajectories using significant plasma proteins (FDR <0.05). **(B)** Pathway enrichment analysis. Pathway direction is the median log2 fold change relative to baseline of significant molecules in each pathway (blue, downregulated; red, upregulated). Dot size represents pathway significance. **(C–F)** Heatmaps representing longitudinal trajectories of plasma proteins in cluster 1 **(C)**, cluster 2 **(D)**, cluster 3 **(E)**, and cluster 4 **(F)**. Protein names are listed on the right (blue, downregulated; red, upregulated).

Cluster analysis identified four clusters of circulating plasma proteins with different longitudinal trajectories. The levels of some plasma proteins decreased after acute heat exposure and did not return to baseline within 24 h (cluster 1). The levels of some plasma proteins increased after acute heat exposure and continued to increase during recovery (cluster 2). There was a delayed increase in the levels of some plasma proteins after acute heat exposure (cluster 3). The levels of some plasma proteins increased after acute heat exposure, and quickly returned to baseline (cluster 4) (Figure 5A). Pathway enrichment analysis was performed on each cluster (Supplementary Figures S3, S4). As depicted in Figure 5B, cluster 1 encompassed proteins associated with the immune-related pathways (immune response, antigen binding etc.), cluster 2 included proteins associated with the coagulation-related pathways (blood coagulation, fibrin clot formation, plasminogen activation), cluster 3 consisted of proteins associated with the cytoskeleton and extracellular matrix (structural constituent of cytoskeleton, extracellular region, extracellular space), and cluster 4 comprised proteins associated with oxygen transport (organic acid binding, hemoglobin complex etc.).

#### 3.4.1 Cluster 1

Cluster 1 was enriched in molecules associated with the innate and humoral immune response (Figure 5C). There was a sharp decrease in the plasma levels of immunoglobulin related fragments (i.e., IGHV3-73, IGLV6-57, IGHV3-38, IGHV3-15, IGHV3-9, IGHV3-43D, IGHV3-43, IGLL1, IGLV3-25, IGHV1-58, IGKV1-27, IGLV7-46, IGKV1D-33, IGKV1-33, IGHV1-45, IGLV2-11, IGKV1-8, FCGR3B, and IGLV3-1) and pattern recognition receptor (i.e., C-type mannose receptor 2). These data indicated that immune function was suppressed immediately by acute heat exposure and did not recovery in short term.

#### 3.4.2 Cluster 2

Cluster 2 was enriched in molecules associated with the coagulation cascade (platelet glycoprotein V, platelet factor 4, coagulation factor VIII, thrombospondin 1, coagulation factor XIII A chain, coagulation factor IX, coagulation factor X, coagulation factor XII, coagulation factor XIII B chain, coagulation factor V, von Willebrand factor), stress related proteins (serine/threonine-protein kinase/endoribonuclease, heat stress protein 90-beta), and glyceraldehyde-3-phosphate dehydrogenase, which is a key glycolysis rate limiting enzyme (Figure 5D). These data implied elevated blood viscosity, risk of disseminated intravascular coagulation (DIC), anaerobic metabolism and the stress response induced by acute heat exposure.

#### 3.4.3 Cluster 3

Cluster 3 was enriched in proteins of the cytoskeleton and components of intercellular junctions (keratin, type II cytoskeletal 2 epidermal, vimentin, coronin-1A, alpha-actinin-1, keratin, type II, cytoskeletal 1, keratin, type I cytoskeletal 10, keratin, type II cytoskeletal 6B, desmoglein-1, keratin, type II cytoskeletal 6C, matrix Gla protein) (Figure 5E). This could be a result from the degradation of cytoskeleton, a visible component diffusely distributed throughout the cell, caused by acute heat exposure. The damaged cytoskeleton elements were latterly released to the plasma and therefore detected in a delayed manner.

#### 3.4.4 Cluster 4

Cluster 4 was enriched in hemoglobin (hemoglobin subunit alpha, hemoglobin subunit beta, hemoglobin subunit delta), carbonic anhydrase (carbonic anhydrase 2, carbonic anhydrase 1), plasminogen, S100A8, the anti-fibrinolytic system (plasminogen activator inhibitor 1) and antioxidants (peroxiredoxin-2, peroxiredoxin-6, kallistatin, superoxide dismutase 1) (Figure 5F). These data implied that hemoglobin and the maintenance of acid–base balance were mobilized under acute heat exposure and the fibrinolytic system and antifibrinolytic system were activated, possibly due to increased coagulation.

### 3.5 Time series system-wide metabolomics data

**Figure 6:**
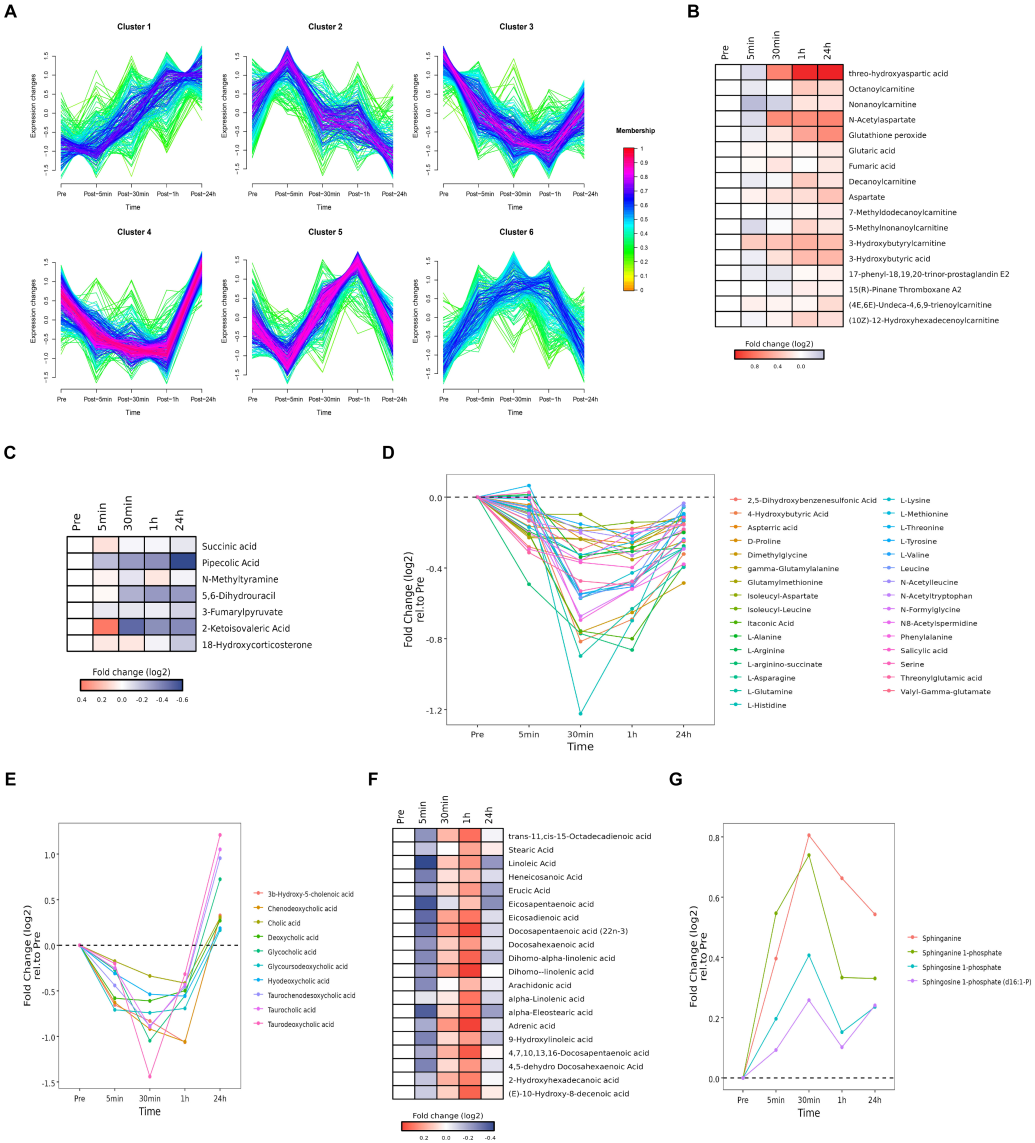
Time series system-wide metabolomics data. **(A)** Clustering of longitudinal trajectories using significant plasma metabolites (FDR <0.05). **(B,C,F)** Heatmaps representing longitudinal trajectories of plasma metabolites in cluster 1 **(B)**, cluster 2 **(C)** and cluster 5 **(F)**. Metabolite names are listed on the right (blue, downregulated; red, upregulated). **(D,E,G)** Time chart representing longitudinal trajectories of plasma metabolites in cluster 3 **(D)**, cluster 4 **(E)**, and cluster 6 **(G)**. Metabolite names are listed on the right.

Cluster analysis identified six clusters of circulating plasma metabolites with different longitudinal trajectories. The levels of some plasma metabolites increased after acute heat exposure and did not return to baseline within 24 h (cluster 1). The levels of some plasma metabolites underwent a transient increase after acute heat exposure but followed by a decrease during recovery (cluster 2). The levels of some plasma metabolites decreased after acute heat exposure and returned to baseline within 24 h (cluster 3, cluster 4). The levels of some plasma metabolites increased after acute heat exposure and returned to baseline within 24 h (cluster 5, cluster 6) (Figure 6A).

Pathway enrichment analysis was performed on each cluster (Supplementary Figure S5). Cluster 1 was associated with alanine, aspartic acid, and glutamic acid metabolism. Cluster 2 was associated with steroid hormone biosynthesis. Cluster 3 was associated with amino acid metabolism. Cluster 4 was associated with cholesterol metabolism. Cluster 5 was associated with the biosynthesis of unsaturated fatty acids. Cluster 6 was associated with sphingolipid metabolism.

#### 3.5.1 Cluster 1

Cluster 1 was enriched in alanine, aspartic acid, and glutamic acid metabolites (i.e., threo-hydroxyaspartic acid, aspartate, *N*-acetylaspartate, fumaric acid, and glutaric acid) and carnitine and acyl carnitine metabolites [i.e., 3-hydroxybutyric acid, (10Z)-12-hydroxyhexadecenoylcarnitine, (4E,6E)-undeca-4,6,9-trienoylcarnitine, 3-hydroxybutyrylcarnitine, 5-methylnonanoylcarnitine, 7-methyldodecanoylcarnitine, decanoylcarnitine, nonanoylcarnitine, octanoylcarnitine] (Figure 6B). Accumulation of carnitine and acyl carnitine metabolites likely reflected disordered fatty acid metabolism. Accumulation of glutathione peroxide reflected oxidative stress. Accumulation of 17-phenyl-18,19,20-trinor-prostaglandin E2 and 15(R)-pinane thromboxane A2 suggested endothelial damage and increased coagulation.

#### 3.5.2 Cluster 2

Cluster 2 was enriched in metabolites associated with tyrosine metabolism (i.e., *N*-methylamine, 3-fumarylpyruvate), pantothenate and CoA biosynthesis (i.e., 5,6-dihydrouracil, 2-ketoisovalic acid), and lysine decomposition (i.e., succinic acid, pipecolic acid) (Figure 6C). These data implied acute heat exposure was associated with the use of amino acids as an energy source. Accumulation of mineralocorticoid (i.e., 18-hydroxycorticosterone carbohydrate) suggested alterations in the regulation of water and salt balance.

#### 3.5.3 Cluster 3

Cluster 3 was enriched in amino acids (i.e., l-tyrosine, l-arginino-succinate, threonylglutamic acid, itaconic acid, *N*-acetyltryptophan, gamma-glutamylalanine, 2,5-dihydroxybenzenesulfonic acid, dimethylglycine, l-arginine, d-proline, isoleucyl-leucine, glutamylmethionine, aspterric acid, l-methionine, *N*-formylglycine, valyl-gamma-glutamate, *N*-acetylleucine, l-valine, 4-hydroxybutyric acid, salicylic acid, l-asparagine, l-glutamine, N8-acetylspermidine, isoleucyl-aspartate, l-histidine, l-lysine, l-threonine, leucine, serine, l-alanine, phenylalanine) (Figure 6D). These data implied acute heat exposure was associated with the use of amino acids as an energy source, suggesting protein supplementation may be helpful after exposure to extreme heat.

#### 3.5.4 Cluster 4

Cluster 4 was enriched in bile acid metabolites, including chenodeoxycholic acid, 3β-hydroxy-5-cholenoic acid, hyodeoxycholic acid, glycoursodeoxycholic acid, cholic acid, deoxycholic acid, deoxycholic acid, glycocholic acid, taurochenodesoxycholic acid, taurocholic acid and taurodeoxycholic acid (Figure 6E). These data implied acute heat exposure was associated with alterations in bile acid metabolism.

#### 3.5.5 Cluster 5

Cluster 5 was enriched in free fatty acids, including dihomo-linolenic acid, dihomo-alpha-linolenic acid, 4,7,10,13,16-docosapentaenoic acid, *trans*-11,*cis*-15-octadecadienoic acid, 4,5-dehydro docosahexaenoic acid, docosahexaenoic acid, alpha-eleostearic acid, docosapentaenoic acid (22n-3), 9-hydroxylinoleic acid, adrenic acid, 2-hydroxyhexadecanoic acid, (E)-10-hydroxy-8-decenoic acid, eicosadienoic acid, erucic acid, linoleic acid, arachidonic acid, heneicosanoic acid, eicosapentaenoic acid, *α*-linolenic acid, and stearic acid (Figure 6F). Accumulation of free fatty acids might be due to the inhibition of heat production and reduce of fatty acid digestion.

#### 3.5.6 Cluster 6

Cluster 6 was enriched with products of sphingomyelin metabolism [i.e., sphinganine, sphinganine 1-phosphate, sphingosine 1-phosphate, sphingosine 1-phosphate (d16:1-P) (Figure 6G)]. Metabolites of sphingomyelin are important signaling molecules, which may be involved in regulating cell proliferation, immune function, inflammatory reactions, and oxidative stress.

### 3.6 Identification of hub molecules involved in the response to acute heat exposure

**Figure 7:**
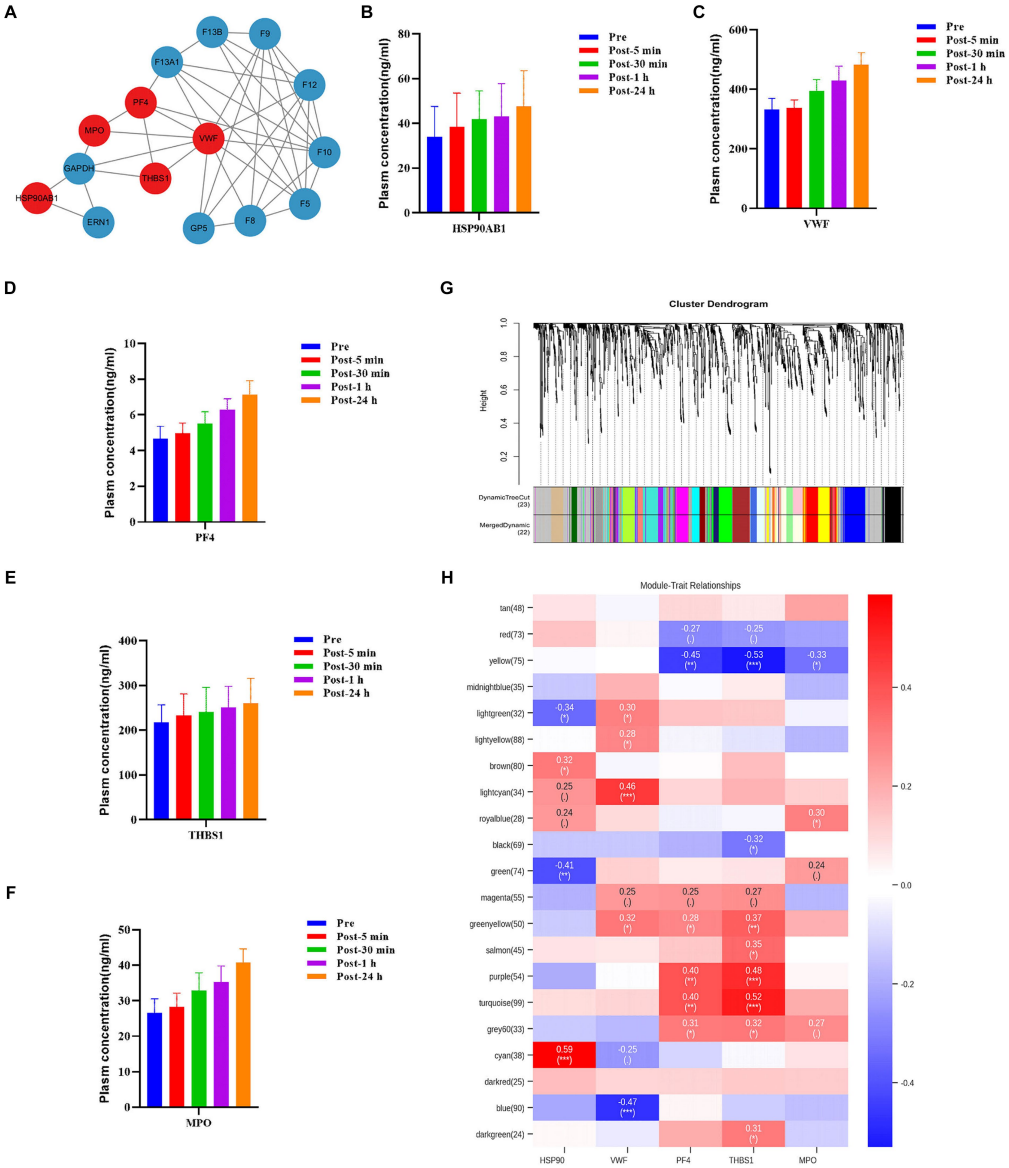
Hub molecules associated with acute heat exposure. **(A)** Protein–protein interaction after acute heat exposure produced by STRING. Line thickness indicates the strength of data support for each interaction. Red colored proteins were associated with acute heat exposure. **(B–E)** Plasma HSP90β, VWF, PF4, THBS1, and MPO levels before and after acute heat exposure were detected by enzyme-linked immunosorbent assay. **(G)** Clustering dendrogram of differentially expressed metabolites. Clustering was based on dissimilarity of metabolites. **(H)** Heatmap of the correlation between metabolites modules and the plasma proteins HSP90, VWF, PF4, THBS1, and MPO.

The levels of some plasma proteins continued to increase after acute heat exposure, implying they are key regulators of heat exposure. To assess the interaction of these plasma proteins and identify potential plasma proteins modulating the response to acute heat exposure, protein–protein interaction analysis was performed. Several important nodes, corresponding to VWF, PF4, THBS1, HSP90AB1, and MPO, were revealed (Figure 7A). These proteins were also validated using ELISA, which showed their levels increased after acute heat exposure (Figures 7B–F and Supplementary Table S3).

Additionally, weighted correlation network analysis measured the co-expression relationship between VWF, PF4, THBS1, HSP90AB1, MPO and plasma metabolites. Hierarchical clustering with dynamic tree cut methods were applied to identify metabolite modules (Figure 7G). Pearson’s correlation coefficient showed HSP90AB1, VWF, PF4 and THBS1 were associated with metabolite modules, identifying HSP90AB1, VWF, PF4 and THBS1 as hub proteins. HSP90AB1 was correlated with the green metabolite module and the cyan metabolite module. VWF was correlated with the lightcyan metabolite module and the blue metabolite module. PF4 was correlated with the yellow metabolite module, the purple metabolite module, and the turquoise metabolite module. THBS1 was correlated with the yellow metabolite module, the purple metabolite module, the turquoise metabolite module, and the green-yellow metabolite module (Figure 7H). KEGG pathway enrichment analysis was performed on the metabolites in the key modules correlated with the hub proteins (Supplementary Figure S6). These modules were enriched in fructose and mannose metabolism, choline metabolism, cholesterol metabolism, histidine metabolism, carbohydrate digestion and absorption, and sphingolipid metabolism. The data implied a regulatory role of HSP90AB1, VWF, PF4 and THBS1 to metabolites in acute heat exposure.

### 3.7 Multi-omics prediction of body temperature and cardiopulmonary parameters

**Figure 8:**
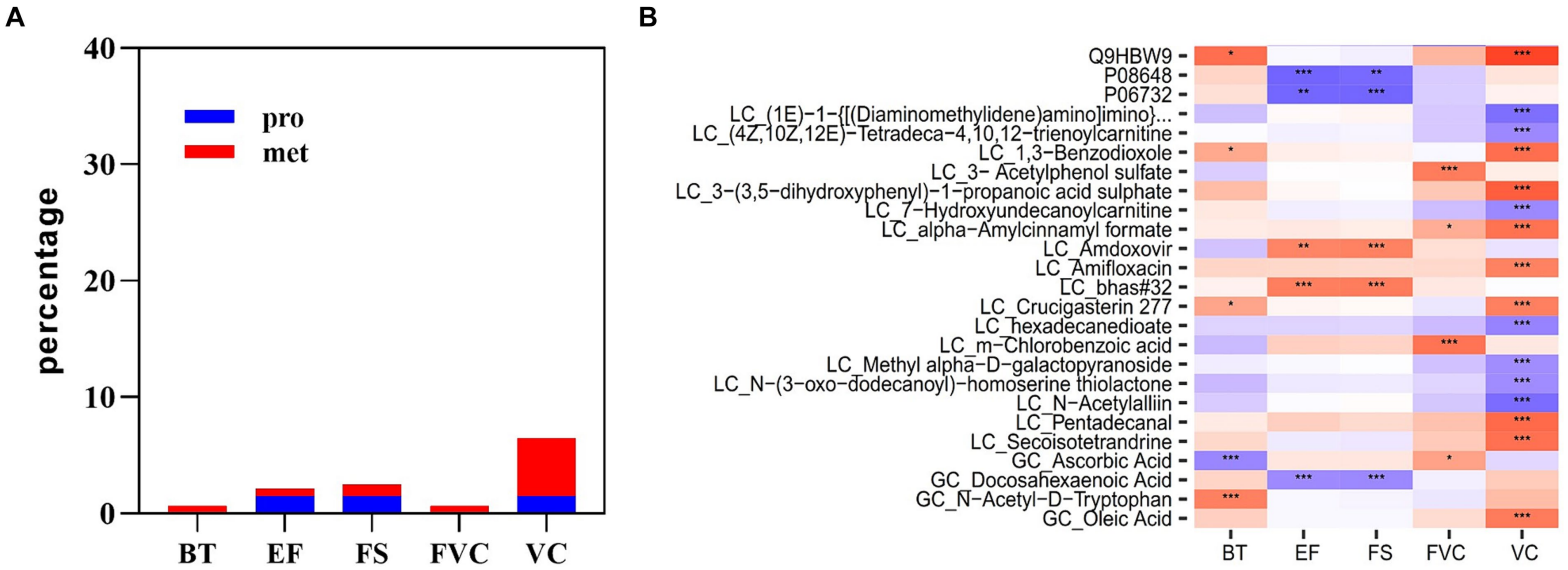
Multi-omics prediction of body temperature and cardiopulmonary function. **(A)** The proportion of plasma proteins and metabolites associated with body temperature and cardiopulmonary parameters. **(B)** Heatmap showing the correlations between plasma analytes and body temperature and cardiopulmonary parameters. * represents *p* < 0.05, ** represents *p* < 0.01 and *** represents *p* < 0.001.

Multi-omics data at the time point immediately after acute heat exposure was used to assess the relevance of clinical data for immediately detecting acute heat exposure. Correlation analysis identified a set of biomarkers that were highly predictive of changes in body temperature, FVC VC, EF, and FS (Figure 8). Body temperature was correlated with metabolites of ascorbic acid and *N*-acetyl-d-tryptophan. FVC was correlated with the metabolites of acetylphenol sulfate and chlorobenzoic acid. VC was correlated with the plasma protein adhesion G protein-coupled receptor L4. EF and FS were correlated with the plasma proteins integrin alpha-5 and creatine kinase M-type, metabolites of amdoxovir and docosahexaenoic acid. These biomarkers have been previously associated with body temperature and the cardiopulmonary system, highlighting their clinical value (27–30).

## 4 Discussion

The increasing global temperatures and incidence of occupational exposure to extreme high temperatures require enhanced understanding of how heat exposure impacts human health (31). This study investigated the short-term tolerance of humans to extreme high temperatures and characterized the detailed series of events that occur in response to acute heat exposure (50°C). Longitudinal multi-omics profiling of plasma components revealed acute heat exposure affected physiological processes in a time-dependent manner. These physiological processes included the immune response, coagulation, acid–base balance, oxidative stress, cytoskeleton maintenance, and energy metabolism. Time series data showed variations in the dynamics of these physiological processes and the molecular changes underlying the response to acute heat exposure. HSP90AB1, VWF, PF4, and THBS1 were identified as key regulators of the physiological processes involved in the response to acute heat exposure. A small number of blood-based analytes were predictors of changes in cardiopulmonary function, with potential for use as a clinical tool to evaluate the health risks of heat exposed populations.

### 4.1 Physiological performance in acute heat exposure

In this study, the physiological performance of the subjects was detected. We found that during heat exposure, subjects exhibited typical physiological reactions, such as increased heart rate, to ensure proper circulation of tissues. However, after heat exposure, subjects suffer some impairment of cardiac function, which may be related with the fluid loss or the impaired vascular function (18, 32). Furthermore, our study revealed a decrease in lung function following heat exposure. This finding aligns with a previous study that found a consistent association between each 1°C increase in lifetime summer mean temperature and a 1.07% decrease in forced vital capacity (FVC) (33). These findings suggested that the subjects included in our study exhibited some adaptive responses to acute heat exposure but experienced a slight decline in their cardiopulmonary function. To mitigate these effects, it is advisable to implement preventive measures such as adequate hydration before, during, and after heat exposure, and monitoring individuals for signs related to heat exposure. Additionally, ensuring proper acclimatization to heat and providing education on heat exposure management can help enhance the resilience of individuals facing acute heat exposure.

### 4.2 Immune response in acute heat exposure

Although there were no significant changes in the vital clinical signs of the subjects after extreme high temperature exposure, the changes in molecular reactions still indicated the potential increased risk after heat exposure. With respect to the immune response, the present study showed that synthesis of immunoglobulin was reduced after heat exposure, indicating the decreased immune function even short time exposure to extreme high temperature. This finding is consistent with previous reports. An investigation of individuals who worked in the foundry showed chronic heat exposure was associated with a decrease in the number of white blood cells (34). Additionally, Chen et al. (35) found the strong associations between heat waves and emergency department visits for intestinal infection. Studies of patients hospitalized for heat stroke during sustained heatwaves have reported a high incidence of urinary, blood or lung infections during recovery (36–38). This phenomenon may be related to abnormal development and decreased number of lymphocytes (39). Findings from a mouse model with heat exposure showed that whole blood collected 30 days after recovery from heat exposure exhibited an immunosuppressive phenotype (40).

### 4.3 Coagulation in acute heat exposure

Coagulation disorders accompany heat-related illness, and severe DIC caused by heatstroke can be fatal (41–43). Findings from the present study imply that coagulation, fibrinolytic and anti-fibrinolytic processes are mobilized in the initial phase of heat exposure. In particular, coagulation-related factors, including VWF, PF4, and THBS1, remained elevated 24 h after acute heat exposure. VWF binds the glycoprotein Iba on platelets causing platelet adhesion, activation and aggregation, which is crucial for hemostasis and thrombosis (44, 45). PF4, also known as chemokine (C-X-C motif) ligand 4, has the ability to bind with negatively charged polyanions such as VWF, resulting in the formation of immune complexes that can contribute to the development of thrombosis (46). THBS1, which is produced by platelets when stimulated by thrombin, plays a role in platelet aggregation (47). The increase in coagulation related factors reflects a hypercoagulant state suggesting that modulating coagulation early after exposure to high temperature may be beneficial for preventing heat-related illness. In previous studies, various anticoagulants, such as antithrombin III, recombinant thrombomodulin and tissue factor pathway inhibitors, had been used to prevent heatstroke (48, 49).

### 4.4 Acid–base balance in acute heat exposure

Acid–base balance is regulated by hemoglobin and carbonic anhydrase levels, which increased in response to acute heat exposure in the present study. Hemoglobin in red blood cells binds oxygen and releases hydrogen ions (50). Carbonic anhydrase catalyzes the reversible hydration of carbon dioxide (CO_2_) to form carbonic acid (H_2_CO_3_) in the lungs, kidneys, and red blood cells (51). Previous studies showed that weekly heat training increases elite cyclists or cross-country skiers’ hemoglobin mass, enhancing their ability to use oxygen (52–54). In the present study, the increases in hemoglobin and carbonic anhydrase were temporary and returned to baseline levels the day after acute heat exposure, indicating that repeated heat exposures are needed to maintain increased levels of hemoglobin and carbonic anhydrase.

### 4.5 Oxidative stress in acute heat exposure

Oxidative damage may be induced by chronic and long-term heat exposure, which enhances GST and GPX enzymatic activity and nitric oxide concentration (55, 56). Consistent with this, the present study showed prooxidants such as MPO and S100A8 were upregulated after acute heat exposure. Interestingly, some antioxidants (peroxiredoxin-2, peroxiredoxin-6, kallistatin, superoxide dismutase 1) were induced to resist harmful effects. Peroxiredoxin-2, peroxiredoxin-6 and superoxide dismutase are antioxidant enzymes that protect organisms from oxidative damage caused by reactive oxygen species (ROS) (57, 58). Kallistatin antagonizes TNF-α-induced oxidative stress, and its active site is crucial for stimulating antioxidant enzyme expression (59). Previous reports showed the negative effects of heat exposure on oxidative status might be alleviated by dietary antioxidant supplementation (60, 61). The present study adds to this growing body of evidence.

### 4.6 Energy metabolism in acute heat exposure

Energy metabolism was altered by acute heat exposure. Levels of circulating plasma metabolites, such as amino acids, free fatty acids, bile acid, carnitine and sphinganine, were changed. Amino acid levels decreased over time as they were catabolized for energy generation. Acylcarnitines and fatty acids accumulated after acute heat exposure. This may be due to the transformation of beige adipocytes into white adipocytes, which can inhibit adipocyte heat production and reduce fatty acid digestion (62). Sphinganine levels increased briefly, confirming the role of sphingosine as a potent regulator of signaling pathways. Levels of the bile acids chenodeoxycholic acid, cholic acid and deoxycholic acid decreased, which might be associated with the decrease in that was observed in high-temperature environments (63).

### 4.7 Potential regulators and biomarkers associated with acute heat exposure

Levels of some circulating plasma proteins (PF4, F13A1, F13B, F9, F12, F10, F5, F8, GP5, VWF, THBS1, MPO, GAPDH, HSP90AB1, and ERN1) continued to increase during the recovery phase after acute heat exposure, implying these proteins are potential regulators of important physiological processes involved in the response to acute heat exposure. Protein–protein interaction and WGCNA analyses identified HSP90AB1, VWF, PF4, and THBS1 as hub proteins. In previous literature, HSP90AB1, VWF, PF4, and THBS1 have been documented to exert substantial influence on various aspects of cell function. HSP90AB1, also known as HSP90β, serves as molecular chaperones to maintain protein stability and has protective roles in response to various kinds of cellular stress, such as heat exposure (64, 65). VWF, PF4, and THBS1 play a critical role in coagulation and regulate immune cells and inflammatory responses (66–68).

After heat exposure, we found that the changes in the levels of certain plasma molecules were significantly correlated with the changes in clinical parameters. Notably, some of these molecules have previously been associated with clinical parameters, suggesting their potential clinical value in predicting functional changes following heat exposure. One of such molecules is *N*-Acetyl-d-Tryptophan, which showed a positive correlation with body temperature after acute heat exposure. This molecule is a derivative of tryptophan and can be metabolized into 5-hydroxytryptamine (5-HT), and activation of 5-HT receptors can lead to an increase in body temperature (69, 70). Furthermore, we observed that the increase of creatine kinase M-type leptin and the decline in docosahexaenoic acid were found to be correlated with the decline in cardiac function after heat exposure. Creatine kinase, an enzyme involved in ATP production and utilization, has been extensively studied as potential risk marker for cardiovascular events (71). Docosahexaenoic acid is an omega-3 fatty acid, which is found to have positive effects on cardiac function and significantly decreases the overall mortality in patients diagnosed with coronary heart disease (72). Additionally, we found adhesion G protein-coupled receptor L4 (ADGRL4) was positively associated with pulmonary function after acute heat exposure. ADGRL4 is an orphan adhesion GPCR expressed in endothelial cells that can induce vascular normalization and immune suppression (73), which may contribute to protecting lung function.

### 4.8 Limitations

This study had some limitations. The sample size was small and did not include females or the older adult. Owing to the safety consideration for subjects, the max tolerance time was not detected, and the relative humidity were not set at a high level, which had obvious effect on human’s tolerance time of extreme high temperature. Therefore, molecular changes associated with life-threatening heatstroke could not be explored under the current experimental setting. Furthermore, while this study provided a comprehensive analysis of molecular changes in response to acute heat exposure, the potential regulators and biomarkers identified require additional validation in larger and more diverse populations. Additionally, although the study identified potential biomarkers predictive of changes in body temperature and cardiopulmonary parameters, the clinical significance and practical applications of these findings are yet to be determined. Further studies are needed to evaluate the usefulness of these biomarkers in real-world settings.

## 5 Conclusion

Overall, this study offered insights into key research blanks in health impact assessment of heat exposure. Longitudinal multi-omics profiling identified thousands of molecules that were affected by acute heat exposure, and time-series clustering and network analysis revealed crosstalk between inflammation, coagulation, immunity, acid–base balance, metabolism, and oxidative stress. Potential regulators and biomarkers for acute heat exposure were revealed. These findings have significant implications for both occupational population exposed to heat and the general population during extreme heat environment. It will help to improve the prediction of short-term health outcomes in acute heat exposure and develop prevention strategies to heat-related injuries and mitigate the adverse effects of heat exposure.

## Data availability statement

The datasets presented in this study can be found in online repositories. The names of the repository/repositories and accession number(s) can be found in the article/Supplementary material.

## Ethics statement

The studies involving humans were approved by The Ethics Review Committee of West China Hospital of Sichuan University. The studies were conducted in accordance with the local legislation and institutional requirements. The participants provided their written informed consent to participate in this study.

## Author contributions

JWe: Conceptualization, Data curation, Formal analysis, Investigation, Validation, Writing – original draft, Writing – review & editing. JC: Data curation, Formal analysis, Investigation, Writing – original draft, Writing – review & editing. LW: Data curation, Formal analysis, Investigation, Methodology, Writing – original draft. CL: Data curation, Methodology, Writing – original draft. YZ: Data curation, Investigation, Writing – original draft. JWu: Conceptualization, Formal analysis, Funding acquisition, Visualization, Writing – review & editing. JL: Conceptualization, Formal analysis, Funding acquisition, Project administration, Supervision, Writing – original draft, Writing – review & editing.
