# Supplementary material for: Dynamic molecular choreography induced by acute heat exposure in human males: a longitudinal multi-omics profiling study
Source: Front Public Health. 2024 May 15;12:1384544. doi: 10.3389/fpubh.2024.1384544 (PMC11135052; doi:10.3389/fpubh.2024.1384544)
Supplement: Supplementary file 1 [file Table_1.DOCX]

**Table S1 The profile of the participants**

| **Name** | **Age(year)** | **Gender** | **Height(cm)** | **Weight(kg)** | **Previous occupational extreme heat exposure(time)** | **Hydration before the exposure(ml)** | **Hydration during the exposure(ml)** | **Hydration** **after the exposure(ml)** |
| --- | --- | --- | --- | --- | --- | --- | --- | --- |
| Long SQ | 21 | male | 175 | 70 | 1 | no | no | 150 |
| Hu LP | 39 | male | 170 | 60 | 4 | no | no | 120 |
| Wang CJ | 32 | male | 181 | 80 | 4 | no | no | 500 |
| Xiao Q | 37 | male | 170 | 74 | 4 | no | no | 200 |
| Long XB | 37 | male | 170 | 70 | 4 | no | no | 160 |
| Liang H | 35 | male | 163 | 62 | 4 | no | no | 100 |
| Zeng ZF | 30 | male | 172 | 70 | 4 | no | no | 200 |
| Zhong GP | 28 | male | 171 | 86 | 4 | no | no | 250 |
| Li Z | 29 | male | 165 | 48 | 3 | no | no | 200 |
| Huang H | 36 | male | 167 | 69 | 3 | no | no | 75 |
| Pan FP | 41 | male | 182 | 78 | 5 | no | no | 500 |
| Wang X | 29 | male | 172 | 70 | 4 | no | no | 500 |
| Zheng FS | 35 | male | 170 | 72 | 3 | no | no | 120 |
| Jiang L | 28 | male | 172 | 57 | 4 | no | no | 250 |
| Li DJ | 31 | male | 167 | 58 | 4 | no | no | 220 |
| Yang GC | 32 | male | 170 | 63 | 2 | no | no | 150 |
| Ma J | 30 | male | 174 | 65 | 4 | no | no | 200 |
| Ye ZJ | 30 | male | 165 | 56 | 4 | no | no | 300 |
| Kuang SJ | 45 | male | 170 | 74 | 4 | no | no | 75 |
| Sun ZS | 25 | male | 175 | 65 | 1 | no | no | 500 |
| Liu F | 34 | male | 168 | 73 | 3 | no | no | 150 |
| He ZG | 35 | male | 168 | 63 | 4 | no | no | 120 |
| Liu LX | 30 | male | 172 | 70 | 3 | no | no | 180 |
| Jiang L | 40 | male | 172 | 71 | 3 | no | no | 250 |

**Table S2 Temperature and cardiopulmonary function changes in response to acute heat exposure**

|  | **Temperature(℃)** | | **EF(%)** | | **FS(%)** | | **FVC(L)** | | **VC(L)** | |
| --- | --- | --- | --- | --- | --- | --- | --- | --- | --- | --- |
|  | **mean** | **p** | **mean** | **p** | **mean** | **p** | **mean** | **p** | **mean** | **p** |
| Pre-heat exposure | 36.63 | ＜0.001 | 68.92 | ＜0.001 | 38.99 | ＜0.001 | 3.253 | ＜0.01 | 3.968 | ＜0.01 |
| Post-heat exposure | 37.08 |  | 62.94 |  | 34.29 |  | 3.168 |  | 3.858 |  |

EF means Ejection fraction, FS means fractional shortening, FVC means forced vital capacity, VC means vital capacity

**Table S3 Hub molecules mesured before and after acute heat exposure**

|  | **HSP90AB1 (ng/ml)** | | | **VWF (ng/ml)** | | | **PF4 (ng/ml)** | | | **THBS1 (ng/ml)** | | | **MPO (ng/ml)** | | |
| --- | --- | --- | --- | --- | --- | --- | --- | --- | --- | --- | --- | --- | --- | --- | --- |
|  | **mean** | **F-test** | | **mean** | **F-test** | | **mean** | **F-test** | | **mean** | **F-test** | | **mean** | **F-test** | |
|  |  | ***F*** | ***p*** |  | ***F*** | ***p*** |  | ***F*** | ***p*** |  | ***F*** | ***p*** |  | ***F*** | ***p*** |
| Pre-heat exposure | 34.06 | 19.68 | ＜0.0001 | 331.7 | 43.44 | ＜0.0001 | 4.668 | 36.22 | ＜0.0001 | 218.0 | 1.712 | 0.1562 | 26.63 | 27.84 | ＜0.0001 |
| Post-5 min | 38.41 |  |  | 338.3 |  |  | 4.978 |  |  | 233.2 |  |  | 28.31 |  |  |
| Post-30 min | 41.91 |  |  | 395.3 |  |  | 5.505 |  |  | 241.0 |  |  | 32.85 |  |  |
| Post-1 h | 43.22 |  |  | 429.3 |  |  | 6.284 |  |  | 251.3 |  |  | 35.24 |  |  |
| Post-24 h | 47.72 |  |  | 483.3 |  |  | 7.132 |  |  | 259.7 |  |  | 40.73 |  |  |

**Figure S1. Urine Changes in Response to Acute Heat Stress**

(A) Urine Specific Gravity. (B) Urine PH Value were detected at pre and post acute heat stress.

**Figure S2. Fatigue and Muscle Strength Changes in Response to Acute Heat Stress**

(A) Subjective Fatigue Score. (B) Reaction Time in Objective Fatigue Test. (C) Error Number in Objective Fatigue Test. (D) Muscle Strength of Left Hand. (E) Muscle Strength of Right Hand were detected at pre and post acute heat stress.

**Figure S3. The Go Enrichment Analysis of Protein Changes in Response to Acute Heat Stress**.

Go enrichment analysis of four main metabolite clusters. Length represented the percent of proteins enriched in pathway.

**Figure S4. The KEGG Enrichment Analysis of Protein Changes in Response to Acute Heat Stress.**

KEGG enrichment analysis of four main metabolite clusters. The dot color represented pathway significance. The dot size represented metabolism pathway number.

**Figure S5. The Enrichment Analysis of Metabolism Changes in Response to Acute Heat Stress**

KEGG enrichment analysis of six main metabolite cluster. The dot color represented pathway significance. The dot size represented number of metabolites enriched in pathway.

**Figure S6. KEGG pathway enrichment analysis of the key modules correlated with the hub proteins**

KEGG enrichment analysis of different modules of metabolites. The dot color represented pathway significance. The dot size represented number of metabolites enriched in pathway.
