# Supplementary figures and images for: Dynamic molecular choreography induced by acute heat exposure in human males: a longitudinal multi-omics profiling study
Source: Front Public Health. 2024 May 15;12:1384544. doi: 10.3389/fpubh.2024.1384544 (PMC11135052; doi:10.3389/fpubh.2024.1384544)

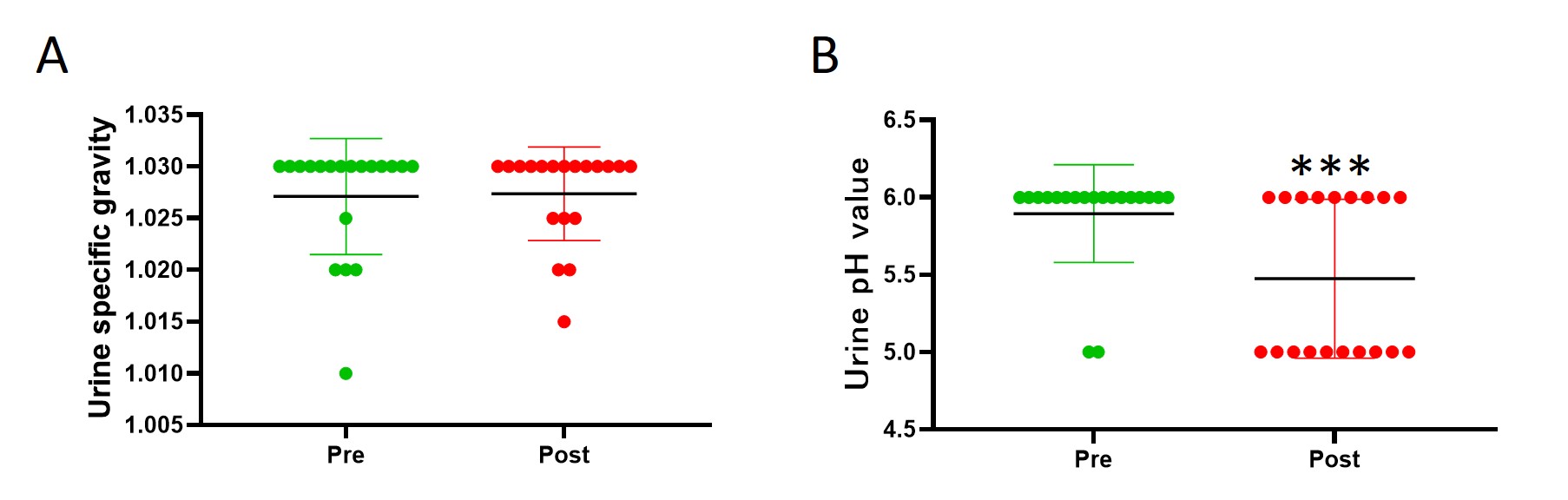

Supplement: Supplementary file 2 [file Image_1.JPEG]

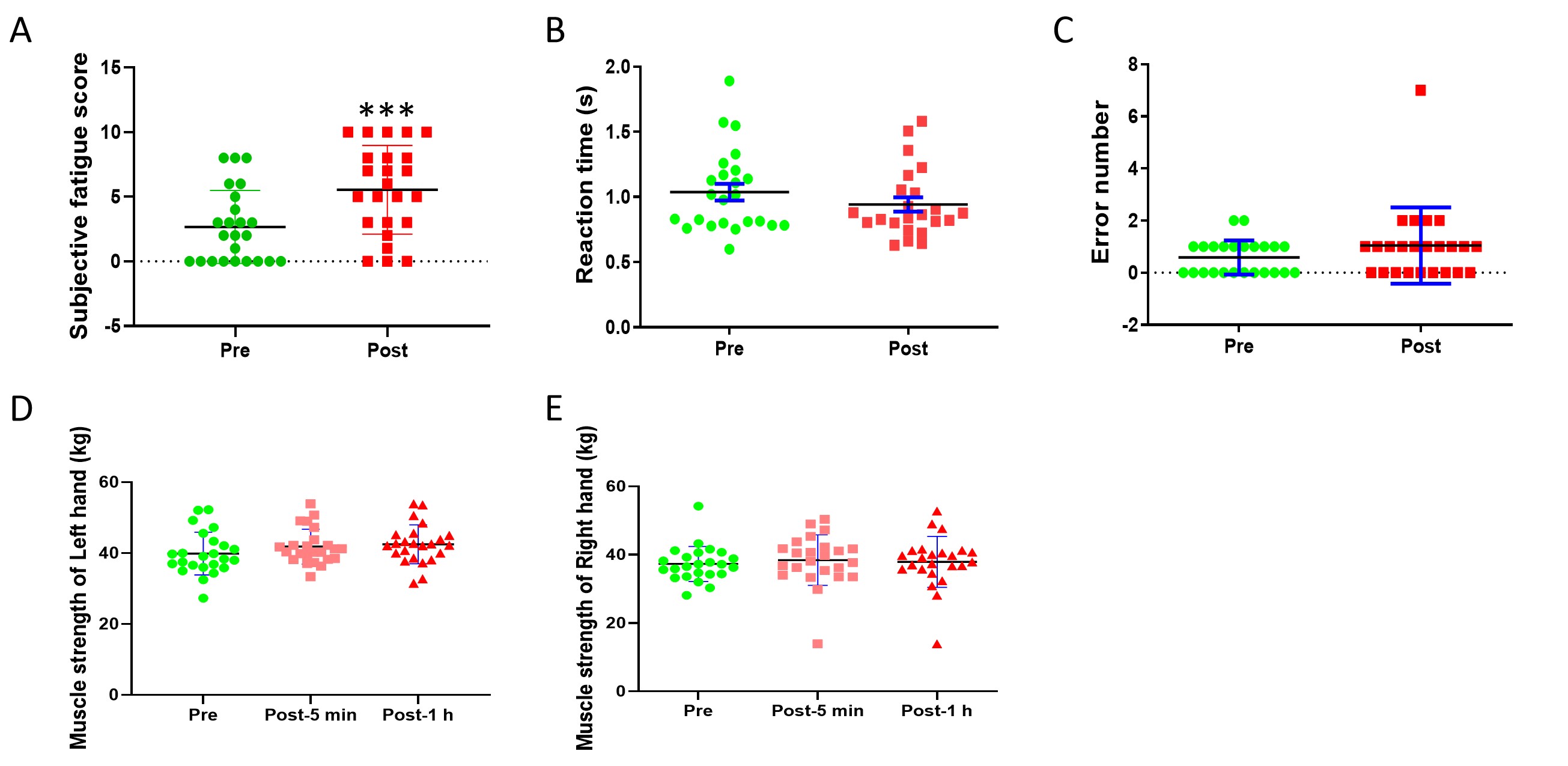

Supplement: Supplementary file 3 [file Image_2.JPEG]

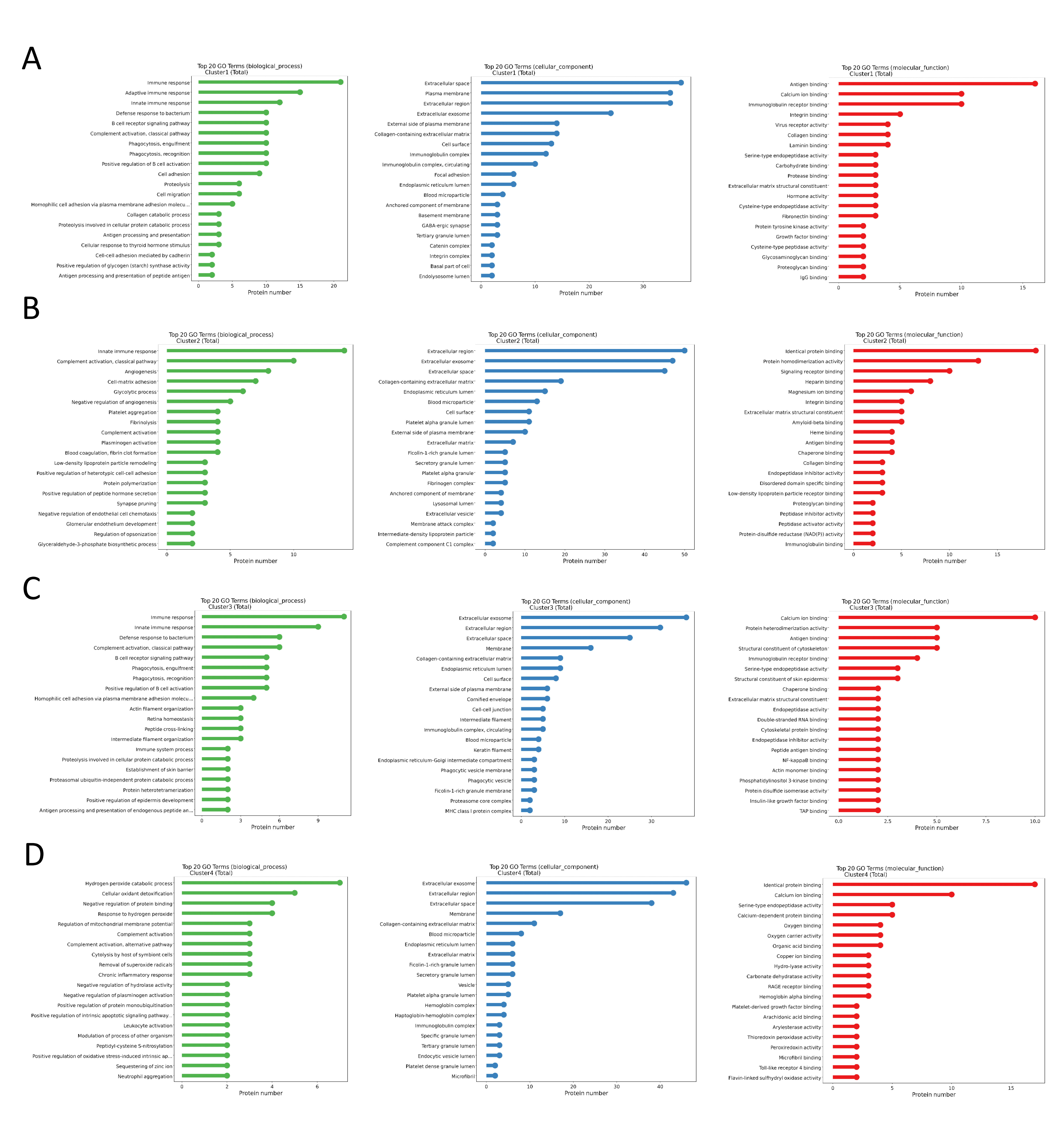

Supplement: Supplementary file 4 [file Image_3.JPEG]

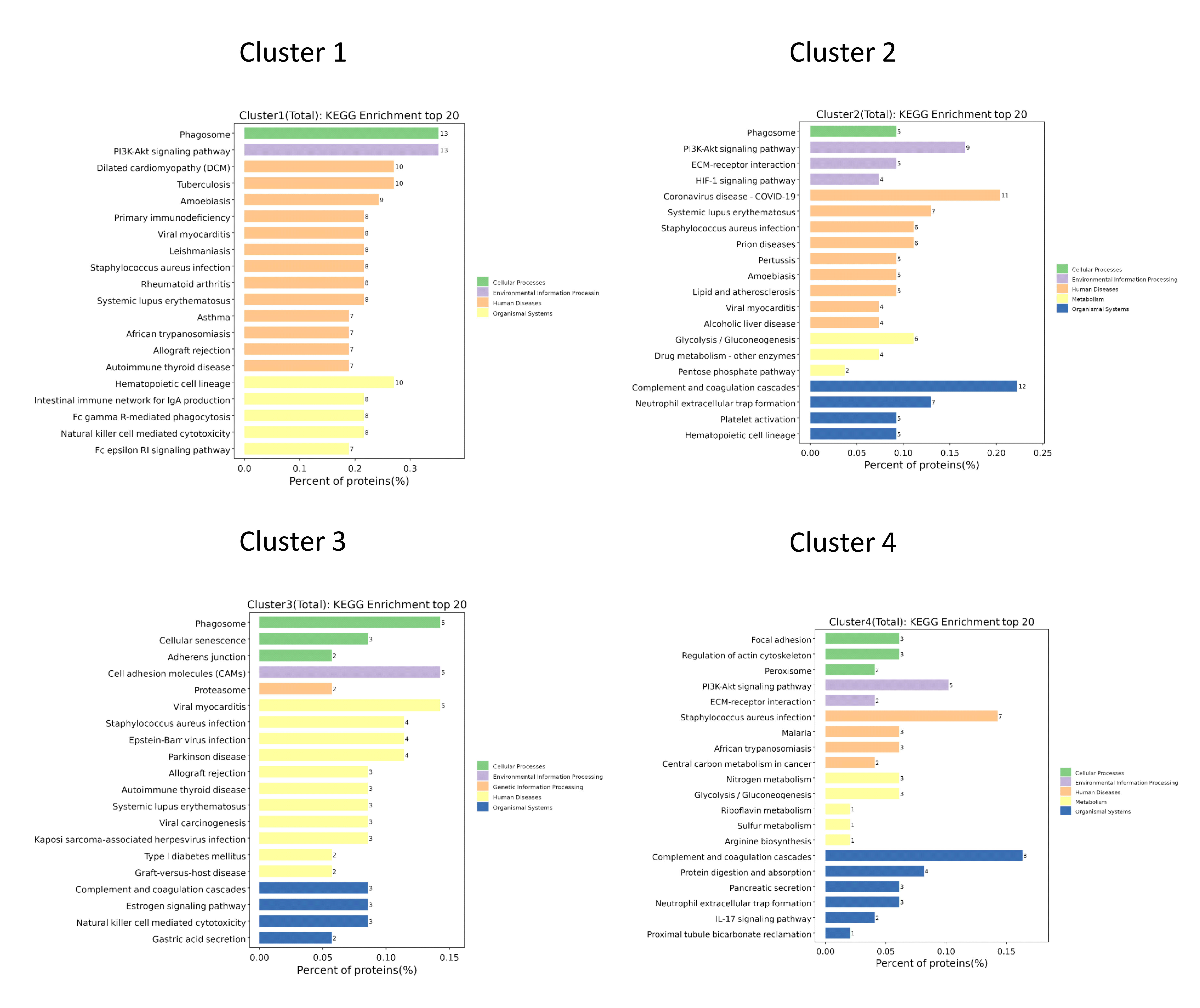

Supplement: Supplementary file 5 [file Image_4.JPEG]
